# Echocardiographic Assessment of the Left Ventricle in Young Prehypertensive Nigerians

**DOI:** 10.7759/cureus.46740

**Published:** 2023-10-09

**Authors:** Isa O Oboirien, Hassan O Yera, Olawale M Akinlade, Oluwamayowa N Omoniyi, Hayatu Umar, Mahmoud U Sani

**Affiliations:** 1 Cardiology, Queen Alexandra Hospital, Portsmouth Hospitals University NHS Trust, Portsmouth, GBR; 2 Internal Medicine, The Shrewsbury and Telford Hospital NHS Trust, Telford, GBR; 3 Cardiology, Dumfries and Galloway Royal Infirmary, Dumfries, GBR; 4 Respiratory Medicine, Royal Papworth Hospital, Cambridge, GBR; 5 Internal Medicine, Usmanu Danfodiyo University Teaching Hospital, Sokoto, NGA; 6 Internal Medicine, Bayero University/Aminu Kano Teaching Hospital, Kano, NGA

**Keywords:** prehypertension, normotensive, nigeria, left ventricular geometry, echocardiography

## Abstract

Background: Prehypertension is associated with an increased risk of cardiovascular morbidity and mortality. This risk could partly be explained by the early compromise in left ventricular (LV) structure and function. This study investigated the LV geometry and function in young black prehypertensive subjects.

Methods and results: This cross-sectional descriptive study was conducted at the Usmanu Danfodiyo University Teaching Hospital, Sokoto, Nigeria. Echocardiography-derived LV geometry and function were assessed using standardized methods. Prehypertensive subjects had higher mean systolic blood pressure (BP) (130.78 ± 3.57 mmHg vs 111.42 ± 3.54 mmHg, *P*<0.001), diastolic BP (79.32 ± 4.13 mmHg vs 66.39 ± 4.42 mmHg, *P*<0.001), body mass index (BMI) (26.24 ± 3.45 kg/m^2 ^vs 22.20 ± 2.21 kg/m^2^, *P*<0.001), waist circumference (WC) (86.93 ± 8.73 cm vs 76.73 ± 6.66 cm, *P*<0.001), fasting blood glucose (FBG) (93.84 ± 7.28 mg/dl vs 90.08 ± 6.26 mg/dl, *P*<0.001), and dyslipidemia (21.5% vs 6%. *P*<0.001) compared to normotensive subjects. LV mass index (LVMI) was greater in prehypertensive subjects compared to normotensive subjects {male (106.84 ± 12.34 g/m^2^ vs 76.07 ± 10.25 g/m^2^, *P*<0.001); female (92.06 ± 8.80 g/m^2^ vs 66.53 ± 7.21 g/m^2^, *P*<0.001)}, with abnormal LV geometry recorded in 17.5%. Linear regression analysis showed that waist circumference, systolic BP, serum creatinine level, and urea level were determinants of LVMI. The prevalence of LV diastolic dysfunction was higher in prehypertensive subjects than in normotensive subjects (14.5% vs. 0.5%, *P*<0.001), with systolic BP {odds ratio (OR) 0.928, confidence interval (CI) 0.834 - 0.969; *P*=0.016)} and diastolic BP (OR 0.832, CI 0.722 - 0.958; *P*=0.011) being independent predictors.

Conclusion: This study showed that prehypertension in young Black subjects was associated with altered LV geometry and impaired diastolic function, and these changes demonstrated linear progression with increasing systolic BP.

## Introduction

Hypertension is a major public health problem, with an estimated global adult prevalence of 26.4% [1]. The relationship between hypertension and the risk of cardiovascular disease events is continuous, consistent, and independent of other risk factors [2]. The risk of developing hypertension is higher in prehypertensive individuals than in normotensive individuals [3]. The overall prevalence of prehypertension is 36%, with a prevalence rate as high as 58% in Nigeria, the most populous Black nation in the world [4,5]. Prehypertension, though a harbinger of hypertension, has been shown to increase the risk of cardiovascular morbidity and mortality [1,6].

Several studies have established the presence of abnormal left ventricular (LV) geometry and function in individuals with prehypertension and hypertension and have also reported a significantly higher LV mass index (LVMI) and abnormal LV diastolic filling in prehypertensive and hypertensive subjects than in normotensive subjects [7-9]. To our knowledge, no study has examined LV geometry and function in young Nigerians with prehypertension. The few studies conducted on LV geometry in Nigeria mostly studied hypertensive subjects, and the results from these studies were correlated to international studies; abnormal LVMI with a predominant pattern of concentric left ventricular hypertrophy (LVH) [10,11]. In addition, the participants in these studies were middle-aged, with a sparse representation of young patients.

Finally, although previous studies have established a correlation between worsening hypertension and increasingly abnormal LV geometry and diastolic function, it is not known if this linear relationship exists with increasing systolic blood pressure (BP) in prehypertensive patients [12,13].

The index study was performed to assess and compare LV geometry and function between normotensive and prehypertensive young adult Nigerians and to examine whether these parameters change with increasing systolic BP in prehypertensive patients.

## Materials and methods

Study population 

The study population consisted of eligible adults attending the general and medical outpatient clinics of Usmanu Danfodiyo University Teaching Hospital (UDUTH), Sokoto, Nigeria. Approval was issued by UDUTH, Sokoto (No. UDUTH/HERC/2013/NO151). Adults aged ≥ 18 years with prehypertension defined as systolic BP between 120 and 139 mmHg and/or diastolic blood pressure between 80 and 89 mmHg provided that the subject had no history of hypertension and had never been on antihypertensive medications were recruited [14]. We excluded subjects <18 years, patients with hypertension defined as sustained systolic blood pressure greater than or equal to 140 mmHg and/or diastolic blood pressure greater than or equal to 90 mmHg or on antihypertensive medications, pre-existing cardiac diseases, chronic kidney disease, pregnant and those on medications that could potentially increase BP such as steroids [14].

Sample size

Using previously reported prevalence of LVH (12.8%); standard normal deviation of 95%; alpha of 5%; and attrition of 20%, the estimated sample size for each arm was 200 subjects [13]. 

Study design

This was a comparative cross-sectional study comprising 200 participants in both arms. Consecutive prehypertensive subjects who met the inclusion criteria were recruited at general, medical, and outpatient clinics. Age- and sex-matched controls made of normotensive volunteers (systolic BP < 120 mmHg and diastolic BP < 80 mmHg) certified by clinical evaluation to be healthy were recruited as controls. All subjects underwent electrocardiography (ECG), transthoracic echocardiography (TTE), and baseline characteristics, including fasting blood glucose, fasting lipid profile, serum urea and creatinine, smoking status, body mass index (BMI), and waist circumference (WC).

Blood pressure measurement

Office BP was measured on the right arm of the patients and controls according to JNC VII recommendations using a validated aneroid sphygmomanometer (Accoson©) [14]. Subjects were in a sitting position, quietly on a chair, with their feet on the ground and arms supported at the level of the heart, and allowed to rest for at least 5 minutes before BP measurement. Subjects and controls abstained from caffeine, exercise, and smoking, at least 30 minutes prior to BP measurement. An appropriately sized cuff with the bladder encircling up to 80% of the arm was used. Systolic BP was determined by radial pulse palpation. The cuff was then inflated to 20-30 mmHg above the level of systolic BP determined by palpation and subsequently deflated at the rate of 2 mmHg/sec. Korotkoff sounds phases I and V were taken as systolic BP and diastolic BP, respectively. Three readings were taken and the average of the last two was taken as the BP. 

Laboratory investigations

Fasting Blood Sugar

Blood (2 ml) was taken from the subjects and transported in a cold container (ice packs in a flask), in a fluoride oxalate bottle. Analysis was done by the glucose oxidase method in the UDUTH Chemical Pathology Laboratory. Subjects fasted overnight for about 8-12 hours prior to sample collection.

Fasting Lipid Profile

Blood (4 ml) was taken from the subjects and transported in a cold container (ice pack in a flask), in a lithium heparin bottle. Analysis was done by the CHOD-PAP enzymatic colorimetric method in the UDUTH Chemical Pathology Laboratory. Subjects fasted overnight for about 8-12 hours prior to sample collection.

*Serum Urea and Creatinine* 

Blood (3 ml) was taken from the subjects and transported in a cold container (ice pack in a flask), in a lithium heparin bottle. Analysis was done in the UDUTH Chemical Pathology Laboratory.

Transthoracic echocardiography

All participants underwent TTE at the Echocardiography Laboratory of UDUTH, Sokoto, using a Sonoscope© SSI-5000 echocardiography machine equipped with a 2.5-MHz transducer. TTE was performed according to the recommendations of the American Society of Echocardiography (ASE) to assess LV geometry and LV function [15].

Left Ventricular Geometry

LV geometry was assessed by measuring the left ventricular internal diameter at end-diastole (LVIDd), left ventricular internal diameter at end-systole (LVIDs), left ventricular posterior wall thickness in diastole (LVPWTd), and interventricular septal thickness in diastole (IVSTd). Left ventricular mass (LVM) was calculated using the Devereux-modified ASE Cube formula [16]. The LVMI was determined by calculating the ratio of LVM to body surface area [17]. Relative wall thickness (RWT) was estimated from LVPWTd and LVIDd [17].

Normal LV geometry was considered as normal LVMI and RWT. Left ventricular concentric remodeling was considered in participants with a normal LVMI and increased RWT (RWT≥0.42). Eccentric LVH was defined as increased LVMI and RWT<0.42, and concentric LVH was defined as increased LVMI and RWT ≥0.42 [17].

Left Ventricular Function

Systolic function: The measurements (LVIDd, LVIDs, and LVPWTd), ejection fraction (EF), and fractional shortening (FS) were calculated using the Teicholz formula. An ejection fraction < 50% constitutes systolic dysfunction [17].

Diastolic function: Transmitral diastolic flow Doppler tracing was obtained from the apical four-chamber view using pulsed Doppler with a sample volume of 1-2 mm placed between the tips of the mitral valve during diastole [17]. The peak early transmitral filling during early diastole (E), peak transmitral atrial filling velocity during late diastole (A), and deceleration time (DT) (time elapsed between peak E velocity and the point where the extrapolated deceleration slope of the E velocity crossed the zero baseline) were determined. Isovolumetric relaxation time (IVRT) was measured by placing a 3-4 mm size sample volume pulsed Doppler at the mitral valve leaflet tips. The transducer beam was angulated toward the left ventricular outflow tract until aortic valve closure appeared above and below the baseline then the IVRT (the period between the end of mitral diastolic flow Doppler tracing and the starting point of aortic flow Doppler tracing) was determined. The Valsalva maneuver was performed to unmask the pseudonormal filling. Patients were instructed to perform a standard Valsalva maneuver by holding a breath and bearing down against a closed glottis at the same time for at least 10 seconds. The early diastolic (E-wave) and atrial contraction (A-wave) Doppler velocities were recorded again in the second phase of the Valsalva maneuver [17].

Pulsed-wave tissue Doppler imaging (TDI) was performed by activating the TDI function using the same echocardiography machine. In the apical 4-chamber view, the TDI cursor sample volume was placed at the lateral side of the mitral annulus. Early (E') and late (A') diastolic mitral annulus velocities and the ratio of the early to late peak velocities (E'/A') were obtained. E/E' was also calculated to assess the left ventricular filling pressure. The mean values of the three different cardiac cycles were obtained.

Statistical analysis

Sociodemographic, clinical, and laboratory characteristics of prehypertensive vs. normotensive subjects were compared using the chi-square test or Fisher exact test for categorical variables. Continuous variables are presented as means and compared using Student’s t-test. The relationship between LVMI and continuous variables of interest, including blood pressure, age, BMI, and waist circumference, was determined using linear regression analysis. Independent determinants of abnormal left ventricular geometry as well as left ventricular dysfunction were determined using multiple logistic regression analysis.

For subgroup analysis, the prehypertensive patients were divided into two groups: group A with systolic BP of 120-129 mmHg, and group B with systolic BP of 130-139 mmHg. LV geometric parameters and function between both groups were compared using Student’s t-test.

Data management and analysis were performed using Statistical Package for Social Sciences (SPSS) version 20.0 (IBM Corp., Armonk, NY) An alpha value of 5% was used for the analysis.

## Results

Baseline characteristics

A total of 200 prehypertensive and normotensive subjects were studied. The prehypertensive and normotensive subjects did not differ in age (28.16 ± 3.09 years vs 28.45 ± 3.27 years, *P*=0.355) and sex (male: female = 1.2:1 in each group) as shown in Table 1. Prehypertensive patients had significantly higher systolic BP {(130.78 ± 3.57 mmHg vs 111.42 ± 3.54 mmHg, *P*<0.001) and diastolic (79.32 ± 4.13 mmHg vs 66.39 ± 4.42 mmHg, *P*<0.001)} than normotensive participants (Table 1). The prehypertensive patients also had higher BMI (26.24 ± 3.45 kg/m2 vs 22.20 ± 2.21 kg/m2, *P*<0.001), WC (86.93 ± 8.73 cm vs 76.73 ± 6.66 cm, *P*<0.001), FBG (93.84 ± 7.28 mg/dl vs 90.08 ± 6.26 mg/dl, *P*<0.001), total cholesterol (169.60 ± 33.17 mg/dl vs 152.62 ± 24.16 mg/dl, *P*<0.001) and LDL cholesterol (99.25 ± 27.94 mg/dl vs 92.29 ± 18.75 mg/dl, *P*=0.004); and significantly lower triglycerides (104.02 ± 19.29 mg/dl vs 94.14 ± 27.75 mg/dl, *P*<0.001) and HDL cholesterol (53.80 ± 3.97 mg/dl vs 51.09 ± 10.43 mg/dl, *P*=0.001) compared to normotensive participants.

**Table 1:** Baseline clinical and laboratory characteristics of the participants BSA, body surface area; BMI, body mass index; SD, standard deviation; WC, waist circumference; SBP, systolic blood pressure; DBP, diastolic blood pressure; FBG, fasting blood glucose; TC, total cholesterol; LDL, low-density lipoprotein; HDL, high-density lipoprotein; TG, triglyceride

Parameters	Prehypertensive (n=200)	Normotensive (n=200)	P value
Age (years), Mean (SD)	28.16 (3.09)	28.45 (3.27)	0.355
Male (%)	110 (55%)	110 (55%)	1.000
Female (%)	90 (45%)	90 (45%)	
Alcohol consumption	10(5)	2(1)	0.019
Weight (Kg), Mean (SD)	73.06(10.59)	61.86(8.06)	<0.001
BSA (per m^2^), Mean (SD)	1.85(0.15)	1.69(0.13)	<0.001
WC (cm), Mean (SD)	86.93(8.73)	76.73(6.66)	<0.001
BMI (Kg/m^2^), Mean (SD)	26.24(3.45)	22.20(2.21)	<0.001
SBP (mmHg), Mean (SD)	130.78(3.57)	111.42(3.54)	<0.001
DBP (mmHg), Mean (SD)	79.32(4.13)	66.39(4.42)	<0.001
Creatinine (mg/dl), Mean (SD)	0.82 (0.19)	0.76 (0.16)	0.003
FBG (mg/dl), Mean (SD)	93.84 (7.28)	90.08 (6.26)	<0.001
TC (mg/dl), Mean (SD)	169.60 (33.17)	152.62 (24.16)	<0.001
LDL (mg/dl), Mean (SD)	99.25 (27.94)	92.29 (18.75)	0.004
HDL (mg/dl), Mean (SD)	51.09 (10.43)	53.80 (3.97)	0.001
TG (mg/dl), Mean (SD)	94.14 (27.75)	104.02 (19.29)	<0.001
Dyslipidaemia (%)	43 (21.5%)	12 (6%)	<0.001

Left ventricular geometric findings

Prehypertensive subjects had significantly higher left ventricular mass index (LVMI) {male (106.84 ± 12.34 g/m2 vs 76.07 ± 10.25 g/m2, *P*<0.001); female (92.06 ± 8.80 g/m2 vs 66.53 ± 7.21 g/m2, *P*<0.001)}, and relative wall thickness (RWT) {male (0.41 ± 0.04 vs 0.38 ± 0.02, *P*<0.001); female (0.40 ± 0.03 vs 0.37 ± 0.01, *P*<0.001)} compared to normotensive controls as presented in Table 2.

**Table 2:** Comparison of left ventricular geometry in normotensive and prehypertensive subjects LVPWd, left ventricular posterior wall diameter at end-diastole; LVIDd, left ventricular internal diameter at end-diastole; LVM, left ventricular mass; LVMI, left ventricular mass index; BSA, body surface area; RWT, regional wall thickness

Parameters	Prehypertensive n=200 (M=110, F=90), Mean (SD)	Normotensive n=200 (M=110, F=90), Mean (SD)	P-value
LVPWd (mm), Male	10.64(0.42)	8.69(0.38)	<0.001
LVPWd (mm), Female	9.68(0.24)	8.02(0.29)	<0.001
LVIDd (mm), Male	49.96(2.04)	45.39(2.28)	<0.001
LVIDd (mm), Female	49.32(1.96)	44.63(1.74)	<0.001
LVM (g), Male	197.66(22.83)	128.56(17.32)	<0.001
LVM (g), Female	170.32(16.29)	112.44(11.62)	<0.001
LVMI (g/BSA), Male	106.84(12.34)	76.07(10.25)	<0.001
LVMI (g/BSA), Female	92.06(8.80)	66.53(7.21)	<0.001
RWT, Male	0.41(0.04)	0.38(0.02)	<0.001
RWT, Female	0.40(0.03)	0.37(0.01)	<0.001

**Figure 1:**
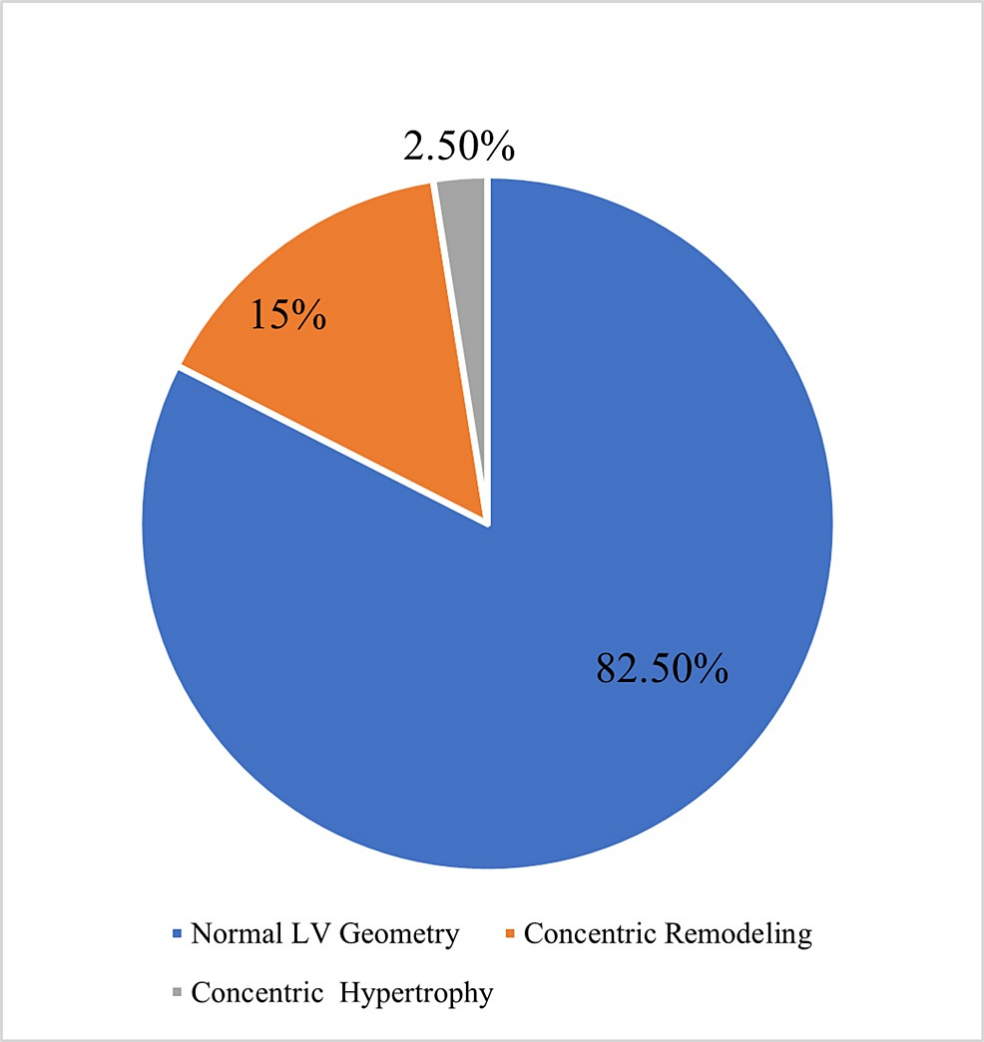
Pie chart showing the pattern LV geometry in prehypertensive individuals LV, left ventricular

The prevalence of abnormal LV geometry was 17.5% among the prehypertensive subjects, whereas all normotensive controls had normal LV geometry. Concentric remodeling (30 {15%}) was the most frequently altered LV geometry pattern observed in prehypertensive subjects, and only five (2.5%) had concentric hypertrophy (Figure 1). None of the prehypertensive subjects showed eccentric hypertrophy.

Left ventricular function findings

**Figure 2:**
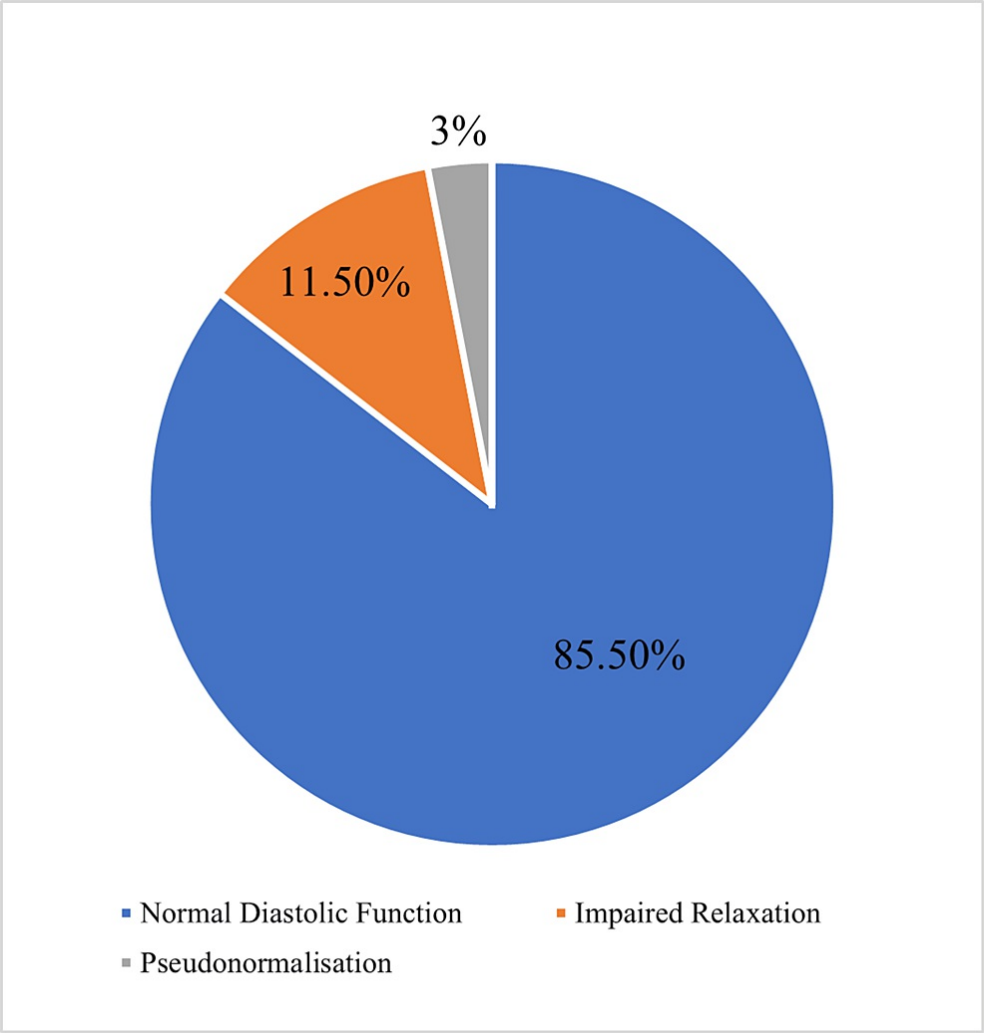
Pie chart showing the pattern of left ventricular diastolic function in prehypertensive individuals

LV systolic function was not significantly different between the prehypertensive and normotensive subjects (69.31 ± 3.66% vs 69.66 ± 3.49%, P=0.328) (Table 3). We observed LV diastolic dysfunction in 14.5% of prehypertensive subjects compared to 0.5% of normotensive subjects. Impaired LV relaxation (11.5%) was the most frequent LV diastolic dysfunction in prehypertensive subjects, with pseudonormalization observed in only 3% of the patients (Figure 2).

**Table 3:** Comparison of left ventricular systolic function parameters between prehypertensive and normotensive subjects LVIDd, left ventricular internal diameter at end-diastole; LVIDs, left ventricular internal diameter at end-systole; LVEF, left ventricular ejection fraction; LVFS, left ventricular fractional shortening; SD, standard deviation

Parameters	Prehypertensive(n=200)	Normotensive (n=200)	P-value
LVIDd (mm), Mean (SD)	49.64(2.01)	45.01(2.05)	<0.001
LVIDs (mm), Mean (SD)	29.87(1.80)	28.29(1.55)	0.002
LVEF, Male (%)	69.24(3.41)	69.18(3.65)	0.909
LVEF, Female (%)	69.39(3.97)	70.23(3.21)	0.188
LVFS Male (%)	32.53(2.53)	32.54(2.53)	0.979
LVFS Female (%)	32.69(2.90)	33.29(2.35)	0.129

None of the prehypertensive participants had a restrictive pattern of LV diastolic dysfunction. Prehypertensive subjects had a significantly lower LV E/A ratio than normotensive subjects (E/A=1.34 ± 0.23 vs 1.45 ± 0.15, P<0.001) while Isovolumetric relaxation time ( VRT) (70.43 ± 13.88 ms vs 61.65 ± 6.65 ms, P<0.001), deceleration time (DT) (188.18 ± 25.33 ms vs 173.18 ± 11.61 ms, P<0.001), and LV filling pressure (E/E') (5.33 ± 0.99 vs 4.96 ± 0.57, P<0.001) were significantly higher in prehypertensive than normotensive subjects as presented in Table 4.

**Table 4:** Comparison of left ventricular diastolic function parameters between prehypertensive and normotensive subjects MV, mitral valve; E Vel, E velocity; A Vel, A velocity; E' Vel, tissue Doppler E' velocity; A' velocity, tissue Doppler A' velocity; IVRT, isovolumetric relaxation time; DT, deceleration time

Parameters	Prehypertensive (n=200)	Normotensive (n=200)	P-value
MV E Vel (cm/s), Mean (SD)	81.03(12.16)	80.96(10.58)	0.949
MV A Vel (cm/s), Mean (SD)	61.64(9.96)	56.44(8.34)	<0.001
MV E/A, Mean (SD)	1.34(0.23)	1.45(0.15)	<0.001
MV E' Vel (cm/s), Mean (SD)	15.83(5.74)	16.37(1.78)	0.205
MV A' Vel (cm/s), Mean (SD)	11.03(4.11)	10.19(1.29)	0.006
MV E/E', Mean, (SD)	5.33(0.99)	4.96(0.57)	<0.001
MV IVRT (ms), Mean (SD)	70.43(13.88)	61.65(6.65)	<0.001
MV DT (ms), Mean (SD)	188.18(25.33)	173.18(11.61)	<0.001
LV Diastolic Dysfunction (%)	29(14.5)	1(0.5)	<0.001

Changes in left ventricular geometry and function with increasing systolic blood pressure in prehypertensive patients

The comparison of LV geometry and function between the prehypertensive subjects with systolic BP (SBP) 130-139 mmHg (group A) and SBP-120-129 mmHg (group B) is shown in Table 5. There was a significant difference in the LVM and LVMI as SBP increased.

**Table 5:** Left ventricular geometry and systolic and diastolic function in prehypertensive subjects: comparison between group A (SBP = 130–139 mmHg) and group B (SBP = 120–129 mmHg) SBP, systolic blood pressure; MV, mitral valve; E Vel, E velocity; A Vel, A velocity; E' Vel, tissue doppler E’ velocity; A' Vel, tissue doppler A’ velocity; IVRT, isovolumetric relaxation time; DT, deceleration time; LV, left ventricular; LVIDd, left ventricular internal diameter at end-diastole; LVIDs, left ventricular internal diameter at end-systole; LVEF, left ventricular ejection fraction; LVFS, left ventricular fractional shortening; LVPWd, left ventricular posterior wall diameter at end-diastole; LVM, left ventricular mass; LVMI, left ventricular mass index; BSA, body surface area; RWT, regional wall thickness

Parameters	Group A (n=86)	Group B (n=114)	P value
LVPWd (mm) Mean (SD)	10.40 (0.38)	9.92 (0.32)	0.002
LVIDd (mm), Mean (SD)	49.80 (2.02)	49.48 (1.97)	0.343
LVM (g), Mean (SD)	190.62 (21.24)	176.95 (18.57)	<0.001
LVMI (g/BSA), Mean (SD)	103.04 (11.48)	95.65 (10.04)	0.004
RWT, Mean (SD)	0.41 (0.04)	0.39 (0.03)	0.001
Abnormal LV geometry (%)	23 (26.8)	12 (10.5)	0.006
LVIDs (mm), Mean (SD)	29.71 (1.78)	30.08 (1.81)	0.141
LVEF (%)	69.01 (3.31)	69.53 (3.91)	0.326
LVFS (%)	32.40 (2.45)	32.75 (2.87)	0.352
MV E Vel (cm/s), Mean (SD)	79.15 (12.93)	82.45 (11.41)	0.058
MV A Vel (cm/s), Mean (SD)	63.04 (10.460	60.58 (9.48)	0.084
MV E/A, Mean (SD)	1.29 (0.26)	1.38 (0.19)	0.004
MV E' Vel (cm/s), Mean (SD)	14.84 (3.32)	16.58 (6.96)	0.033
MV A' Vel (cm/s), Mean (SD)	11.09 (2.24)	10.99 (5.10)	0.898
MV E/E', Mean (SD)	5.46 (0.95)	5.22 (1.01)	0.087
MV IVRT (ms), Mean (SD)	74.59 (16.27)	67.28 (10.81)	<0.001
MV DT (ms), Mean (SD)	195.52 (30.37)	182.63 (19.09)	<0.001
LV diastolic dysfunction (%)	20 (23.2)	9 (7.9)	0.260

Determinant of left ventricular mass index and predictors of left ventricular diastolic dysfunction

Table 6 shows that waist circumference (P=0.009), systolic BP (P<0.001), urea (P=0.031), and creatinine (P=0.012) were significant determinants of an increased left ventricular mass index in prehypertensive subjects. With respect to predictors of LV diastolic dysfunction, systolic BP (OR 0.928, CI 0.834 - 0.969; P=0.016) and diastolic BP (OR 0.832, CI 0.722 - 0.958; P=0.011) were identified as independent predictors of LV diastolic dysfunction in prehypertensive subjects (Table 7).

**Table 6:** Determinants of LVMI FBG, fasting blood glucose; T Chol, total cholesterol; LDL, low density lipoprotein; HDL, high density lipoprotein; SBP, systolic blood pressure; DBP, diastolic blood pressure; CI, confidence interval; BMI, body mass index; WC, waist circumference

Variable	Left ventricular mass index (LVMI)				
	B	Beta	T	P value	CI
Age	-0.131	-0.41	-1.018	0.309	-0.368 – 0.107
WC	0.238	0.220	2.632	0.009	0.60 – 0.415
BMI	-0.381	-0.134	-1.595	0.112	-0.850 – 0.089
SBP	0.568	0.583	7.384	<0.001	0417 – 0.719
DBP	-0.053	-0.041	-0.543	0.588	-0.244 – 0.138
Urea	-1.111	-0.089	-2.159	0.031	-2.122 – -0.99
Creatinine	6.093	0.106	2.537	0.012	1.371 – 10.814
FBG	1.323	0.051	1.283	0.200	-0.704 – 3.350
T Chol	-0.010	-0.030	-0.381	0.703	-0.061 – 0.041
LDL Chol	0.043	0.103	1.349	0.178	-0.020 – 0.106
HDL Chol	-0.075	-0.060	-1.083	0.280	-0.020 – 0.061
Triglyceride	0.027	0.066	1.265	0.207	-0.015 – 0.069

**Table 7:** Independent predictors of left ventricular diastolic dysfunction T Chol, total cholesterol; LDL, low density lipoprotein; HDL, high density lipoprotein; SBP, systolic blood pressure; DBP, diastolic blood pressure; FBG, fasting blood glucose; WC, waist circumference; OR, odds ratio; CI, confidence interval; BMI, body mass index

Variables	Left ventricular diastolic dysfunction			
	B	Wald	P value	OR (CI)
Age	0.085	1.224	0.269	0.918 (0.790-1.068)
WC	0.093	3.720	0.047	0.902 (0.829-0.993)
BMI	0.154	1.517	0.218	1.167 (0.913-1.491)
SBP	0.179	5.904	0.016	0.928 (0.834-0.969)
DBP	0.184	6.494	0 .011	0.832 (0.722-0.958)
Urea	-0.206	0.478	0.489	0.814 (0.454-1.460)
Creatinine	-0.838	0.361	0.548	0.433 (0.122-6.665)
FBG	-0.833	1.657	0.198	0.435 (0.122-1.545)
T Chol	-0.013	1.033	0.310	0.987 (0.962-1.012)
LDL Chol	0.029	3.460	0.063	1.029 (0.998-1.061)
HDL Chol	0.009	0.082	0.775	1.009 (0.951-1.070)

## Discussion

This study compared the clinical characteristics, LV geometry, and LV function of prehypertensive and normotensive young adult Nigerians. We found that prehypertensive subjects tended to have a higher BMI, waist circumference, impaired fasting blood glucose, and dyslipidaemia than normotensive subjects. We also report that prehypertensive young adults, unlike normotensive subjects, had a higher incidence of LVH on ECG and abnormal indices of LV geometry and diastolic dysfunction on echocardiographic assessment. Interestingly, we noted that the incidence of these abnormal findings was continuous and increased with increasing systolic BP in patients with prehypertension. Our analysis identified waist circumference, systolic BP, and diastolic BP as independent predictors of abnormal LV geometry. This study suggests that prehypertension is not only associated with abnormal LV geometry and function but that abnormalities worsen with an increase in systolic BP.

Our findings from baseline characteristics in prehypertensive subjects corroborate those of other studies from the USA and Taiwan [18,19]. Prehypertensive individuals had high BMI, waist circumference, and body surface area. However, the difference in the BMI was not statistically significant. We found that prehypertensive individuals were at substantial risk of cardiometabolic diseases as they had significantly higher fasting blood glucose and dyslipidemia levels than normotensive subjects, a finding similar to that of a previous study conducted in Nigeria [5,20].

Our study showed that the LVMI was significantly higher in prehypertensive subjects than in normotensive subjects. This correlates with the work of Ale et al. [10] in Nigeria and other studies [21,22], including the Strong Heart Study [7]. The findings of a significantly higher left ventricular internal diameter in diastole (LVIDd), left ventricular posterior wall in diastole (LVPWd), and relative wall thickness (RWT) in prehypertensive individuals in the current study corroborate the ARIC (Atherosclerosis Risk in Communities) study [23] where LVIDd and LVPWd were significantly higher in prehypertensive subjects compared to normotensive controls amongst Americans. These observations were the same as those reported in previous studies [13,18,19].

The prevalence of abnormal left ventricular geometry in prehypertensive subjects in this study was 17.5%. This is consistent with the findings of a study conducted in Kano [24], Nigeria, with a prevalence of 14%, and Korea [13], where a prevalence of 18.9% was reported. The prevalence of abnormal left ventricular geometry in newly diagnosed hypertensive individuals in Northwest and Southwest Nigeria was 76% and 78.2%, respectively [25,26]. Two different LV geometric patterns were observed in this study. Concentric remodeling was the most common altered LV geometry pattern and was observed in 30 (15%) prehypertensive subjects, while concentric hypertrophy was present in five (2.5%) of the same group. No eccentric hypertrophy pattern was observed. These findings contrast that of Drukteinis et al., who in a study of prehypertensive American Indians, reported eccentric hypertrophy as the commonest abnormal left ventricular geometry followed by concentric remodeling [18]. This variation may be attributed to racial and ethnic differences. Furthermore, as age is known to be a determinant of LVH, the younger age of the participants in this study may explain the finding [27].

The most common left ventricular geometric pattern in newly diagnosed hypertensive individuals in Nigeria has been reported to be concentric hypertrophy [11,25]. In the hypertensive population, the risk of cardiovascular events in LVH is higher with concentric hypertrophy than with concentric remodeling [27]. In the Strong Heart Study [7], the progression from prehypertension to sustained hypertension was predicted by both baseline systolic BP and LVM. The probability of developing incident hypertension increased by 36% for each LVMI standard deviation (SD) of LVMI.

An interesting finding from our study was that we demonstrated a gradient of increase in these indices for LVMI in prehypertensive subjects with a systolic BP between 121 and 129 mmHg compared to those with a systolic BP between 130 and 139 mmHg. Although studies have demonstrated a gradient of increase in the frequencies of altered LVH from prehypertension to hypertension [12,13,28], this gradient of change has not been previously documented. Previous studies have shown that LVH is influenced by increasing BP in hypertensive subjects [12,28], this might not be a surprising finding. In addition, compared to prehypertensive subjects with systolic BP between 121 and 129 mmHg, those with sustolic BP between 130 and 139 mmHg had a significantly lower mitral E/A ratio, and a significantly higher tissue Doppler mitral E' velocity, IVRT, and DT.

The left ventricular ejection fraction and fractional shortening were not significantly different between the prehypertensive and normotensive subjects in the current study. Drukteinis et al. also observed similar findings amongst American Indians [18]. However, Dogru et al. in a study in Turkey, showed that normotensive individuals had significantly higher left ventricular fractional shortening than their prehypertensive counterparts but there was no significant difference in their ejection fraction [28]. The lack of difference in systolic function between prehypertensive and normotensive subjects in our study does not however indicate that myocardial contractility may not be compromised.

The prevalence rates of left ventricular diastolic dysfunction (LVDD) in prehypertension and normotension in this study were 14.5% and 0.5%, respectively. In contrast, Santos et al. reported LVDD prevalence of 59% and 44% in prehypertensive and normotensive individuals respectively [23]. This is likely due to differences in the age groups studied, as aging is a documented determinant of LV diastolic dysfunction [18]. Santos et al. studied the elderly population (prehypertensive - 75.4 ± 4.8 years, normotensive - 74.4 ± 4.8 years) while the current study population is younger (prehypertensive - 28.2 ± 3.1 years, normotensive - 28.5 ± 3.3 years) [23].

The E/E' ratio in prehypertensive individuals in this study, was significantly higher than normotensive controls (5.33 ± 0.99 vs 4.96 ± 0.57, *P*<0.001). This correlates with the findings from the Monica/Kora Augsburg study, where E/E' was higher in the prehypertensive group (9.1 vs 8.5, *P*=0.031) [29]. The IVRT and DT were also significantly higher in prehypertensive subjects in the current study. Though IVRT and DT were higher in prehypertensive subjects in the strong heart study, the differences were not statistically significant [7]. Ethnic differences could account for this variation in result.

The mechanism underlying diastolic dysfunction in young normotensive subjects is not clear. It is proposed that changes at the cellular or interstitial level could be responsible for diastolic function abnormalities even in the absence of clear cardiac remodeling [22].

In the current study, impaired LV relaxation and pseudonormalization were observed in 11.5% and 3% of prehypertensive individuals, respectively. A restrictive pattern of LV diastolic dysfunction was not observed among prehypertensive subjects probably because this pattern of diastolic dysfunction is associated with associated with hypertensive heart disease [11,25].

Strengths and limitations

There are several strengths in our study. It demonstrated that there is a linear relationship between LV geometry, LV function, and rising systolic BP. Findings from our study add to the evidence that early treatment may prevent the development of abnormal left ventricular geometry. As this was a cross-sectional study, it is unclear how rapidly the progression occurs within the prehypertensive blood pressure ranges and to hypertension. This relationship between rising BP and the development of abnormal LV geometry and function, and the potential for reversal will need to be studied in a prospective study. Lastly, echocardiography has the limitation of intra-observer variability which might have affected the results of this study. 

## Conclusions

Our study demonstrated that cardiovascular risk factors (obesity, dyslipidemia, impaired fasting blood sugar) were significantly higher in prehypertensive individuals. It also demonstrated altered left ventricular geometry and left ventricular diastolic dysfunction in prehypertension. Concentric remodeling was the commonest pattern of altered left ventricular geometry, while impaired relaxation was the commonest form of left ventricular (LV) diastolic dysfunction. Systolic blood pressure and waist circumference were independent determinants of left ventricular mass index and diastolic dysfunction.

We recommend that prehypertensive individuals be closely monitored and commence early lifestyle modification to reduce the risk of developing LV diastolic dysfunction and LV hypertrophy. The influence of altered LV geometry and LV diastolic dysfunction in the progression from prehypertension to hypertension as well as their consequences need to be determined in a longitudinal study as the findings from such studies may influence the future determination of the cut-off point of hypertension. Lastly, a multicentre large population study is required to determine the risk-benefit of drug treatment of prehypertension.
